# Multivisceral resection of advanced colon and rectal cancer: a prospective multicenter observational study with propensity score analysis of the morbidity, mortality, and survival

**DOI:** 10.1515/iss-2023-0027

**Published:** 2023-11-27

**Authors:** Michael Arndt, Hans Lippert, Roland S. Croner, Frank Meyer, Ronny Otto, Karsten Ridwelski

**Affiliations:** Institute for Quality Assurance in Operative Medicine, Otto-von-Guericke University at Magdeburg, Magdeburg, Germany; Department of General and Abdominal Surgery, Municipal Hospital (“Klinikum Magdeburg GmbH”), Magdeburg, Germany; Department of General, Abdominal, Vascular and Transplant Surgery, Otto-von-Guericke University at Magdeburg with University Hospital, Magdeburg, Germany

**Keywords:** colon cancer (colon CA), rectal cancer (rectal CA), multivisceral resection (MVR), “propensity-score” analysis, early postoperative outcome, long-term oncological/oncosurgical outcome

## Abstract

**Objectives:**

In the surgical treatment of colorectal carcinoma (CRC), 1 in 10 patients has a peritumorous adhesion or tumor infiltration in the adjacent tissue or organs. Accordingly, multivisceral resection (MVR) must be performed in these patients. This prospective multicenter observational study aimed to analyze the possible differences between non-multivisceral resection (nMVR) and MVR in terms of early postoperative and long-term oncological treatment outcomes. We also aimed to determine the factors influencing overall survival.

**Methods:**

The data of 25,321 patients from 364 hospitals who had undergone surgery for CRC (the Union for International Cancer Control stages I–III) during a defined period were evaluated. MVR was defined as (partial) resection of the tumor-bearing organ along with resection of the adherent and adjacent organs or tissues. In addition to the patients’ personal, diagnosis (tumor findings), and therapy data, demographic data were also recorded and the early postoperative outcome was determined. Furthermore, the long-term survival of each patient was investigated, and a “matched-pair” analysis was performed.

**Results:**

From 2008 to 2015, the MVR rates were 9.9 % (n=1,551) for colon cancer (colon CA) and 10.6 % (n=1,027) for rectal cancer (rectal CA). CRC was more common in men (colon CA: 53.4 %; rectal CA: 62.0 %) than in women; all MVR groups had high proportions of women (53.6 % vs. 55.2 %; pairs of values in previously mentioned order). Resection of another organ frequently occurred (75.6 % vs. 63.7 %). The MVR group had a high prevalence of intraoperative (5.8 %; 12.1 %) and postoperative surgical complications (30.8 % vs. 36.4 %; each p<0.001). Wound infections (colon CA: 7.1 %) and anastomotic insufficiencies (rectal CA: 8.3 %) frequently occurred after MVR. The morbidity rates of the MVR groups were also determined (43.7 % vs. 47.2 %). The hospital mortality rates were 4.9 % in the colon CA-related MVR group and 3.8 % in the rectal CA-related MVR group and were significantly increased compared with those of the nMVR group (both p<0.001). Results of the matched-pair analysis showed that the morbidity rates in both MVR groups (colon CA: 42.9 % vs. 34.3 %; rectal CA: 46.3 % vs. 37.2 %; each p<0.001) were significantly increased. The hospital lethality rate tended to increase in the colon CA-related MVR group (4.8 % vs. 3.7 %; p=0.084), while it significantly increased in the rectal CA-related MVR group (3.4 % vs. 3.0 %; p=0.005). Moreover, the 5-year (yr) overall survival rates were 53.9 % (nMVR: 69.5 %; p<0.001) in the colon CA group and 56.8 % (nMVR: 69.4 %; p<0.001) in the rectal CA group. Comparison of individual T stages (MVR vs. nMVR) showed no significant differences in the survival outcomes (p<0.05); however, according to the matched-pair analysis, a significant difference was observed in the survival outcomes of those with pT4 colon CA (40.6 % vs. 50.2 %; p=0.017). By contrast, the local recurrence rates after MVR were not significantly different (7.0 % vs. 5.8 %; both p>0.05). The risk factors common to both tumor types were advanced age (>79 yr), pT stage, sex, and morbidity (each hazard ratio: >1; p<0.05).

**Conclusions:**

MVR allows curation by R0 resection with adequate long-term survival. For colon or rectal CA, MVR tended to be associated with reduced 5-year overall survival rates (significant only for pT4 colon CA based on the MPA results), as well as, with a significant increase in morbidity rates in both tumor entities. In the overall data, MVR was associated with significant increases in hospital lethality rates, as indicated by the matched-pair analysis (significant only for rectal CA).

## Introduction

Considering the statistical data, it is evident that colorectal carcinoma (CRC) is listed as one of the most common malignant tumors [1, 2]. At the time of diagnosis, a significant proportion of malignant tumors of the colorectum are in the advanced stage (pT3/pT4) without the presence of hematogenous distant metastasis [3, 4]. Thus, intraoperatively, a tumor conglomerate is detected in 1 in 10 patients, the surgical removal of which requires multivisceral resection (MVR). Here, the differences between peritumorous inflammatory malignant adhesion (pT1–pT3) and infiltration (pT4) must be distinguished. Since, in both cases, surgical separation of the tumor conglomerates would lead to a dramatic reduction in overall survival, MVR is always indicated [5], [6], [7], [8], [9].

However, do the survival rates achieved through this method justify the, partially, high morbidity and mortality rates?

This study aimed to evaluate the early postoperative and oncologic/oncosurgical long-term outcome of MVR of colon or rectal cancer (colon CA, rectal CA).

## Patients and methods

Over a defined period of time, all consecutive patients with histologically confirmed colon and rectal CA were included and analyzed on a topic-specific basis. The primary data were obtained from the prospective multicenter observational and quality assurance studies on colon and rectal CA of the “AN-Institut für Qualitätssicherung in der operativen Medizin gGmbH” at the Otto-von-Guericke-University of Magdeburg (Germany).

Data of patients with histologically confirmed primary colon or rectal CA who had been treated surgically were obtained from 364 participating hospitals.

Data were collected for each entity using standardized questionnaires and documented in a computer-based registry. In addition to information on diagnosis and therapy, the demographic data and early postoperative events (determined based on the general and specific complication rates with their specific content points as parameters of morbidity as well as hospital lethality) were recorded. Furthermore, long-term survival was investigated, characterized by 5-year-overall survival, 5-year tumor-free survival, and 5-year-local recurrence rate.

Tumor recurrence was defined as local recurrence (recurrence of the disease within the original tumor bed) or the occurrence of distant metastases.

Patients aged 30 years and older and those with the Union for International Cancer Control (UICC) stages I–III were included in the study. Patients who had undergone palliative procedures alone and those with hereditary cancer syndromes and carcinomas resulting from chronic intestinal diseases were excluded.

Further methodological details have already been sufficiently published several times by the study group [10], [11], [12].

Differences were observed between colon CA and rectal CA as well as between MVR and non-multivisceral resection (nMVR) groups.

### Statistics

Descriptive statistics were used to evaluate the patient-, tumor-, surgery-/therapy-specific primary data of the identified patient.

Continuous variables, such as the age and periods were expressed as mean, standard deviation, and median. Meanwhile, categorical variables were expressed as absolute and relative frequencies. The chi-square test was used to identify independent variables; if the sample size was <5, Fisher’s exact test was used. The *U* test was used to analyze the systematic difference among continuous variables (for example, sex). A p value of < 0.05 implied that the “null hypothesis” was rejected, and the result was considered significant.

The oncological long-term parameters were evaluated by conducting a Kaplan–Meier survival analysis. The log-rank test was used to compare the survival differences among the study groups. The 5-year survival rate, median survival time, and the corresponding 95-% confidence interval (CI) were obtained. Additional multivariable tests were performed using the backward Cox regression analysis. The hazard ratio (HR) and corresponding 95-% CI were obtained.

In order to increase the value of the statistical statement, a matched-pair analysis was also performed. In this analysis, patients from the MVR group were matched with those from the nMVR group by “propensity score matching”. The combinations were made by matching the patients by sex, age, tumor-node-metastasis stage, and tumor location.

Statistical analysis was performed using IBM^®^ SPSS^®^ Statistics software (version 24.0; copyright 1989–2016, SPSS Inc., Chicago/IL, U.S.A.).

## Results

From 2008 to 2015, in 15,604 patients with colon CA, 1,551 MVR procedures (9.9 %) were performed. Of the 9,717 rectal CA patients who underwent surgery, MVR was required in 1,027 patients (10.6 %) (Table 1).

**Table 1:** Description of the patient population, tumor and node status, proportion of curative and palliative patients, and number of resected organs; consideration of tumor and resection type.

		Colon						Rectum					
		MVR		nMVR		In total		MVR		nMVR		In total	
		n	%	n	%	n	p	n	%	n	%	n	p
**Gender**													

Men		720	46.4	7,605	54.1	8,325	<0.001	460	44.8	5,562	64.0	6,022	<0.001
Women		831	53.6	6,448	45.9	7,279		567	55.2	3,128	36.0	3,695	

**Age**													

Mean ± S_D_^a^		71.2 ± 11.5		71.5 ± 10.8			0.001	68.8 ± 11.0		68.4 ± 11.0			0.725

**Invasion depth**													

pT1		49	3.1	1,622	11.5	1,671	<0.001	72	7.0	1,124	12.9	1,196	<0.001
pT2		132	8.5	2,763	19.6	2,895		175	17.0	2,903	33.3	3,078	
pT3		631	40.6	8,263	58.6	8,894		453	44.1	4,351	49.9	4,804	
pT4		744	47.8	1,455	10.3	2,199		328	31.9	337	3.9	665	

**Lymph nodes**													

pN0		949	61.0	9,363	66.4	10,312	<0.001	637	62.0	5,803	66.6	6,440	0.006
pN1		355	22.8	3,100	22.0	3,455		241	23.4	1,884	21.6	2,125	
pN2		252	16.2	1,640	11.6	1,892		150	14.6	1,028	11.8	1,178	

**Therapy intention**													

Curative		1,442	93.1	13,872	98.8	15,314	<0.001	930	90.5	8,511	97.9	9,441	<0.001
Palliative		107	6.9	174	1.2	281		98	9.5	182	2.1	280	

**Number of organ resections**													

	1	1,176	75.6				<0.001	654	63.7				<0.001
	2	302	19.4					253	24.6				
≥	3	78	5.0					120	11.7				

MVR, multivisceral resection; nMVR, non-multivisceral resection; S_D_, standard deviation.

With regard to the results of the matched pair analysis, 1,410 colon CA patients (2,820 valid data) and 822 rectal CA patients (1,644 valid data) were determined.

### Patient, demographic, tumor, and therapeutic characteristics

The mean age of the colon CA-related MVR group was 71.2 ± 11.5 years, while that of the comparison group (nMVR) was 71.5 ± 10.8 years (p<0.001). After comparing both MVR groups, we found that patients with rectal CA were significantly younger (p<0.001). The mean ages of patients with this tumor type were 68.8 ± 11.0 (MVR) and 68.4 ± 11.0 years (nMVR) (p=0.725).

Men were more frequently affected (colon: 53.4 %; rectum: 62.0 %) than women; on the contrary, the proportion of women was high in all MVR groups (colon: 53.6 %; rectum: 55.2 %).

**Figure 1:**
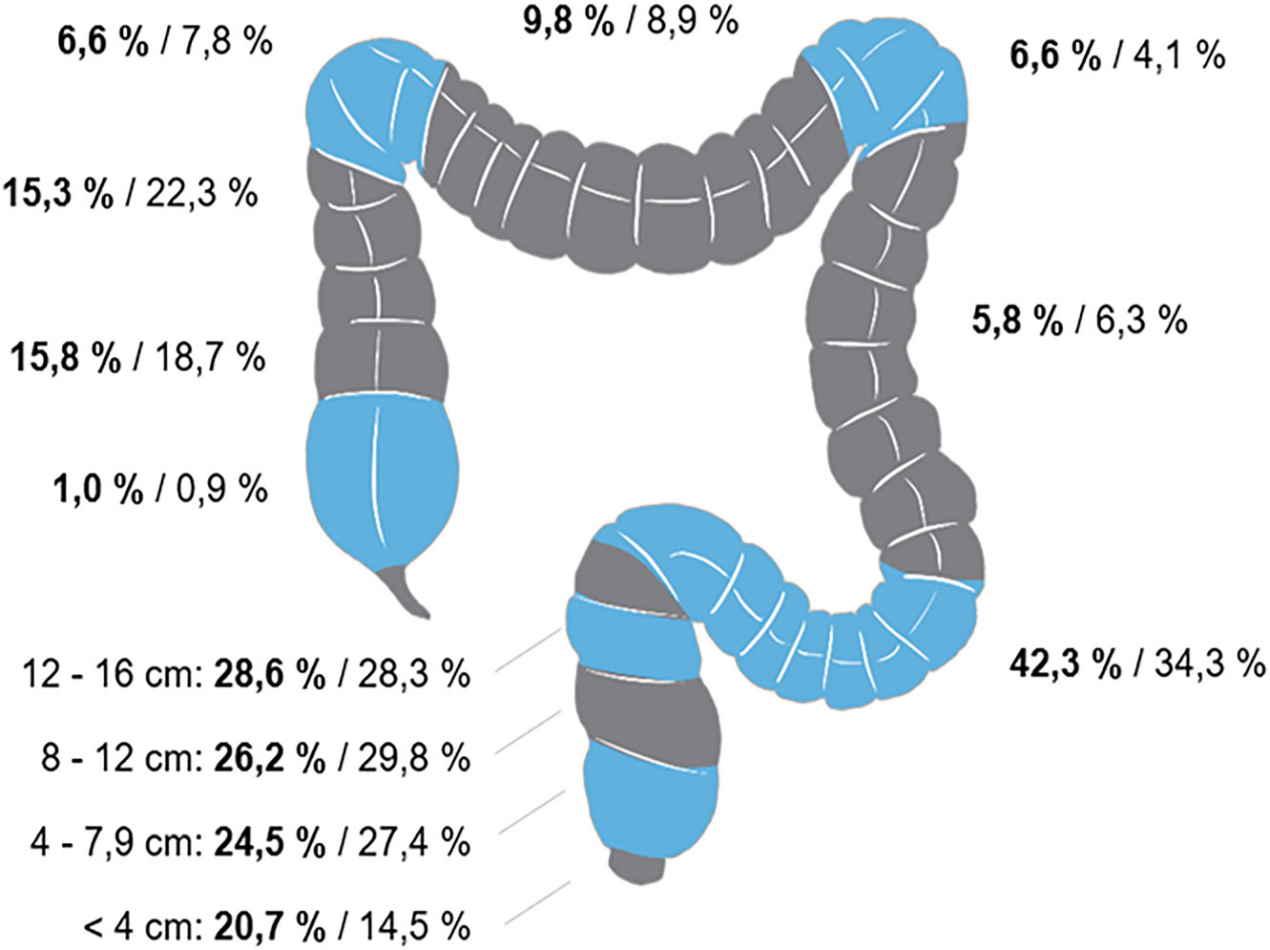
Tumor localizations in colon and rectal cancer. Order of numbers MVR/nMVR, respectively, multivisceral resection/non-multivisceral resection.

A tumor was detected and MVR was performed in each intestinal segment (Figure 1). In the MVR groups, colon CA frequently occurred in the sigmoid colon (MVR: 42.3 %; nMVR: 34.3 %), while rectal CA frequently occurred in the upper segment (12–16 cm ab ano; 28.6 %). By contrast, the middle segment of the rectum (8–12 cm ab ano; 29.8 %) dominated in the nMVR group.

In the colon CA group, MVR was frequently performed in patients with pT4 (47.8 %) and pT3 (40.6 %) tumors. In the rectal CA group, MVR was frequently performed in patients with pT3 (44.1 %) and pT4 (31.9 %) tumors. In both the colon and rectal CA groups, MVR was frequently performed in patients with pT2 and pT1 carcinomas (Table 1).

As the number of organs to be resected increased, the frequency of MVR decreased within both tumor entity samples. The three most frequently resected organs in the colon CA group were the small intestine (28.5 %), abdominal wall (16.4 %), and adnexa (13.7 %). In rectal CA, the adnexa (23.0 %), uterus (16.8 %), and colonic parts (15.5 %) were the most commonly resected additional organs.

Overall, infiltration was detected in 41.4 % of colon CA patients and in 28.3 % of rectal CA patients who underwent MVR. In MVR patients with pT4 tumors, the colon CA group had an infiltration rate of 82.2 %, while the rectal CA group had an infiltration rate of 85.5 %. In the nMVR groups, 41.3 % of colon CA patients and 25.1 % of rectal CA patients with stage pT4 did not undergo MVR despite the existing infiltration (Table 2).

**Table 2:** Detected malignant infiltrations for colon cancer in absolute (n) and relative (%) frequencies by organ and resection type.

	Colon						Rectum				
		MVR		nMVR			MVR		nMVR		
	Infiltration	n	%	n	%	p	n	%	n	%	p
pT1–4	Yes	632	41.4	481	3.5	<0.001	288	28.3	102	1.2	<0.001
	No	896	58.6	13,403	96.5		728	71.7	8,531	98.8	
pT4	Yes	602	82.2	423	30.1	<0.001	277	85.5	93	28.1	<0.001
	No	130	17.8	983	69.9		47	14.5	238	71.9	

MVR, multivisceral resection; nMVR, non-multivisceral resection.

In approximately 90 % of patients who had undergone MVR, an R0 status was achieved, although the relative proportion was significantly greater in the nMVR groups (colon CA: 92.3 % vs. 98.6 %; p<0.001/rectal CA: 89.2 % vs. 97.6 %; p<0.001).

### Morbidity

Intraoperative complications rarely occurred in the colon CA patients who underwent nMVR (2.3 %). In the MVR group, 5.8 % of the patients developed intraoperative complications (p<0.001). The difference was further pronounced in the rectal CA group (4.6 % vs. 12.1 %; p<0.001). When comparing the postoperative surgical complication rates of nMVR (colon CA: 22.4 %; rectal CA: 28.8 %) and MVR groups (colon CA: 30.8 %; rectal CA: 36.4 %), MVR was associated with significantly higher rates (p<0.001 in each case). Wound infection (7.1 %), anastomosis insufficiency, and bowel atony (6.0 % each) were the most common surgical complications in the colon CA-related MVR group. In the rectal CA group, anastomosis insufficiency (8.3 %), infected sacral cavity (6.1 %), and wound infection requiring surgery (5.3 %) were the most common surgical complications. Pneumonia (colon CA: 7.4 %) and urinary tract infection (rectal CA: 8.7 %) were the most common general complications after MVR. When comparing both types of resection, a significant difference was observed in the overall morbidity (colon CA: 43.7 % vs. 32.3 %; rectal CA: 47.2 % vs. 36.3 %; both p<0.001). In both MVR groups, morbidity was found to be at a lower level using matched-pair analysis, but also with a significant difference (colon CA: 42.9 % vs. 34.3 %/rectal CA: 46.3 % vs. 37.2 %; p<0.001 in each case) (Table 3).

**Table 3:** Complication rates, morbidity, and lethality according to the tumor entity and resection type; additional indication of the results of the matched-pair analysis.

	Colon						Rectum					
	MVR		nMVR		In total		MVR		nMVR		In total	
	n	%	n	%	n	p	n	%	n	%	n	p
Complications												

Intraoperative	91	5.8	326	2.3	417	<0.001	123	12.1	402	4.6	525	<0.001

Postoperative												

General	425	27.3	2,568	18.2	2,993	<0.001	260	25.5	1,397	16.2	1,657	<0.001
Surgical	479	30.8	3,157	22.4	3,636		372	36.4	2,497	28.8	2,869	

Morbidity												

Total		43.7		32.3		<0.001		47.2		36.3		<0.001
MPA		42.9		34.3		<0.001		46.3		37.2		<0.001

Lethality												

Total	75	4.9	418	3.0	493	<0.001	39	3.8	229	2.6	268	<0.001
MPA	67	4.8	52	3.7	119	0.084	28	3.4	25	3.0	53	0.005

MVR, multivisceral resection; nMVR, non-multivisceral resection; MPA, matched-pair analysis.

### Lethality

The MVR of colon CA showed a hospital lethality of 4.9 % (nMVR: 3.0 %; p<0.001), while the MVR of rectal CA had a hospital lethality of 3.8 % (nMVR: 2.6 %; p<0.001). Values continued to trend elevated in colon CA (MVR) in matched-pair analysis, but without statistical significance (4.8 % vs. 3.7 %; p=0.084). For rectal CA, hospital mortality significantly increased in the MVR group as indicated in the results of the overall data analysis (3.4 % vs. 3.0 %; p=0.005) (Table 3).

### Survival

**Figure 2:**
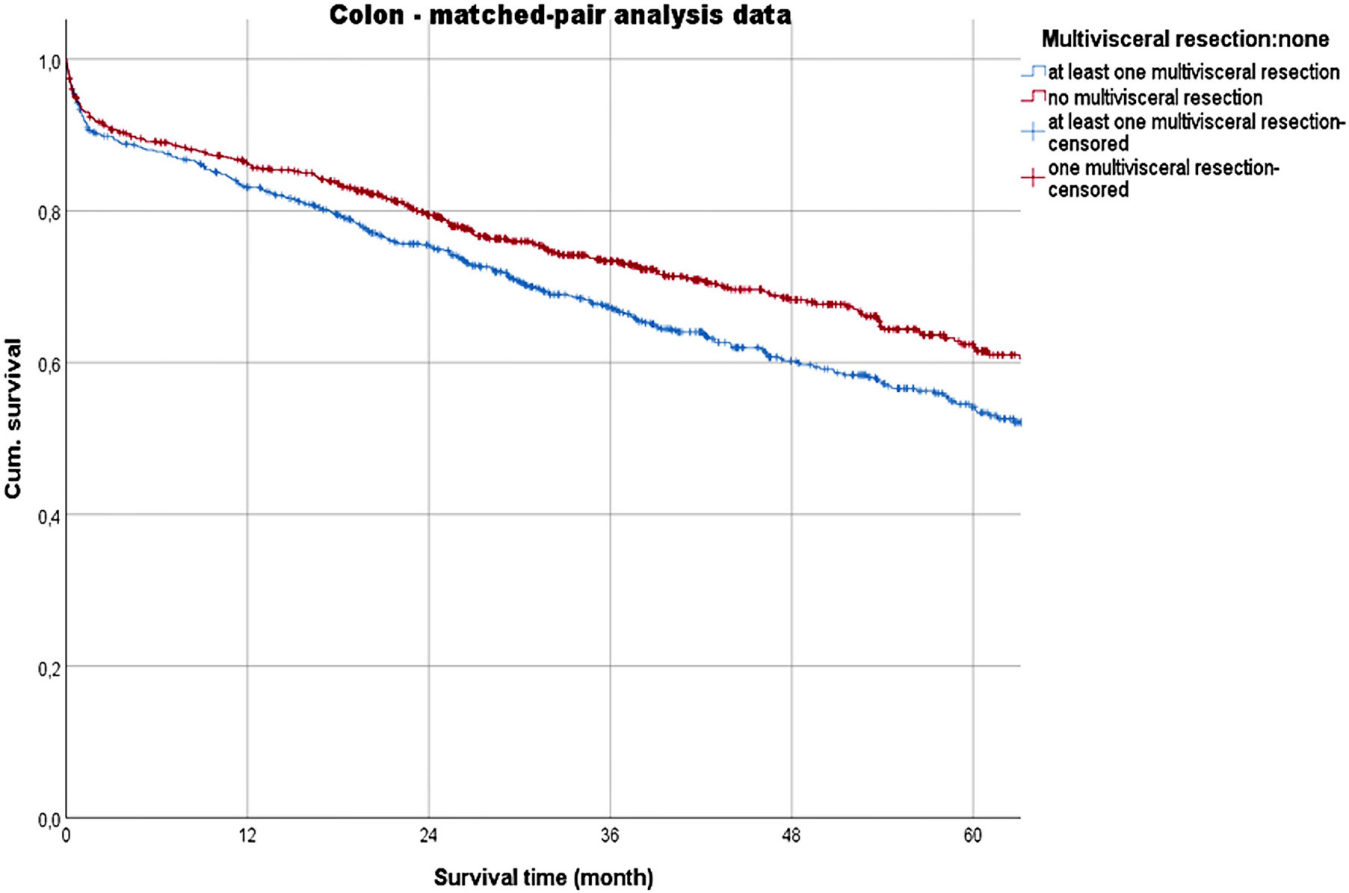
Survival time (matched-pair analysis data) of the multivisceral resection and non-multivisceral resection groups with colon cancer.

**Figure 3:**
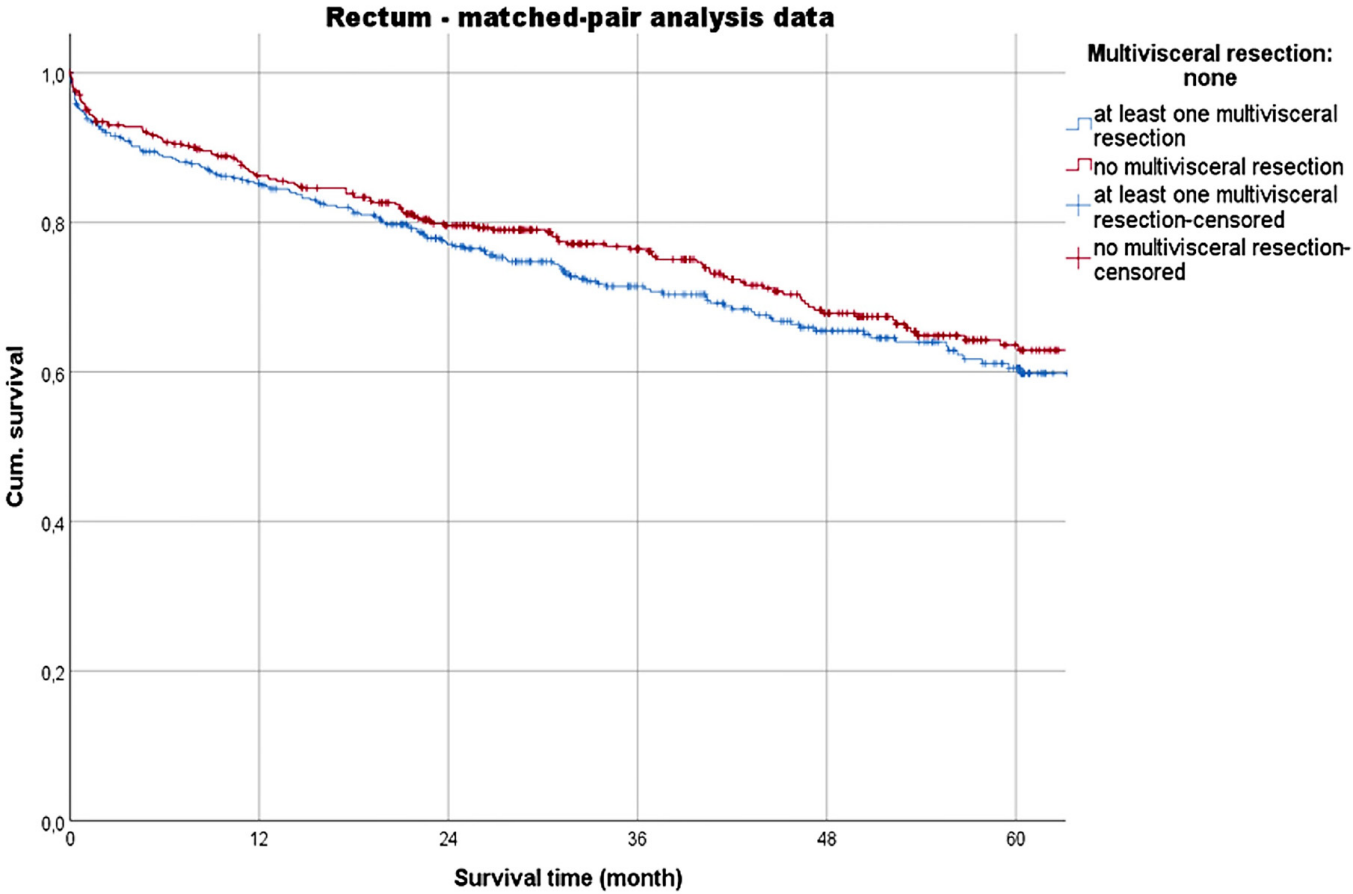
Survival time (matched-pair analysis data) of the multivisceral resection and non-multivisceral resection groups with rectal cancer.

With regard to the overall survival across stages, the 5-year-survival rates (5-YSR) of the MVR groups were reduced (p<0.001). In a head-to-head comparison of the MVR groups, the 5-YSR was low for colon CA (53.9 % vs. 56.8 % [rectal CA]). The matched-pair analysis showed slightly higher rates (colon CA: 54.1 %; rectal CA: 60.5 %) (Table 4, Figures 2 and 3).

**Table 4:** Survival, disease-free survival, and local recurrence rates according to the tumor entity and resection type with results of log-rank tests; results of the matched-pair analysis noted in parentheses.

Colon							Rectum				
Survival											
	Group	Med. survival		5-YSR		p	Med. survival		5-YSR		p
Total	MVR	66.8	(66.8)	53.9	(54.1)	<0.001 (0.004)	84.6	(88.7)	56.8	(60.5)	<0.001 (0.189)
	nMVR	–	(91.1)	69.5	(62.4)		106.6	(–)	69.4	(63.6)	

pT status											

pT2	MVR	81.2	(–)	65.6	(68.4)	0.057 (0.096)	88.7	(88.7)	78.2	(78.2)	0.378 (0.372)
	nMVR	–	(–)	77.6	(77.4)		–	(–)	76.8	(75.6)	
pT3	MVR	–	(–)	63.5	(63.8)	0.266 (0.563)	–	(–)	59.4	(59.1)	0.361 (0.842)
	nMVR	99.1	(–)	67.6	(68.9)		93.3	(–)	62.4	(57.5)	
pT4	MVR	46.2	(46.0)	42.1	(40.6)	0.074 (0.017)	43.2	(41.5)	38.4	(35.8)	0.798 (0.945)
	nMVR	52.4	(63.7)	46.7	(50.2)		42.0	(40.1)	44.3	(43.2)	

R status											

R0	MVR	74.7	(74.7)	55.6	(55.4)	<0.001 (<0.001)	84.6	(88.7)	58.0	(62.2)	0.022 (0.005)
R+	MVR	29.2	(21.3)	31.7	(25.1)		42.0	(41.4)	45.4	(33.3)	

**Disease-free survival (DFS)**											
	Group	Med. survival		5-DFS		p	Med. survival		5-DFS		p

Total	MVR	51.9	(53.8)	46.8	(47.5)	<0.001	54.2	(67.3)	47.6	(51.8)	<0.001 (0.144)
	nMVR	–	(87.4)	66.0	(57.4)	(<0.001)	93.3	(–)	62.9	(55.6)	
**Local recurrence rate (LRR)**											
	Group			5-Y-LRR		p			5-Y-LRR		p

Total	MVR			8.1	(7.0)	<0.001			7.3	(5.8)	0.025
	nMVR			2.8	(4.6)	(0.203)			4.9	(5.8)	(0.587)

MVR, multivisceral resection; nMVR, non-multivisceral resection; 5-YSR, 5-year-overall survival rate; 5-Y-LRR, 5-year local recurrence rate; Med. survival, median survival (in months).

With regard to the individual infiltration depths, the 5-YSR of colon and rectal CA patients with pT2 to pT4 disease were reduced. Comparisons of pT stages (MVR vs. nMVR) revealed no significant differences (p>0.05), but the 5-YSR were low in the MVR group and high in the rectal CA group. In the matched-pair analysis, a significant difference was found in the 5-YSR of pT4 colon CA patients (40.6 % vs. 50.2 %; p=0.017).

The 5-year-local recurrence rate (LRR) of colon CA-related (MVR: 8.1 % vs. nMVR: 2.8 %; p<0.001) and rectal CA-related MVR patients (MVR: 7.3 % vs. nMVR: 4.9 %; p=0.025) were significantly increased. According to the matched-pair analysis, the values were identical in rectal CA patients (5.8 % in each case; p=0.587). In colon CA, the 5-year-LRR of the MVR group tended to be increased (7.0 % vs. 4.6 %; p=0.203) (Table 4).

With regard to the 5-year disease-free survival (DFS), local recurrence, distant metastasis, and death were counted as events. The 5-year DFS were lower in both MVR groups (colon CA: 46.8 % vs. 66.0 %/rectal CA: 47.6 % vs. 62.9 %; both p<0.001). The matched-pair analysis showed prolonged 5-year DFS in the nMVR groups (47.5 % vs. 57.4 % [p<0.001] in colon CA; 51.8 % vs. 55.6 % in rectal CA; p=0.144).

### Factors influencing overall survival

In colon CA, five significant prognostic factors of overall survival were identified (Table 5). These included morbidity (HR: 2.17; 95-% CI: 1.69–2.78; p<0.001) and age, among others. Compared with those aged below 50 years, patients with colonic CA had a more than fourfold increased risk of death after reaching the age of 79 years (HR: 4.45; 95-% CI: 1.98–9.99; p<0.001).

**Table 5:** Multivariate analysis by matched-pair analysis listing selected factors influencing overall survival in colon and rectal cancer.

Prognostic factors of overall survival						
	Factor type	Parameter	HR	95-% Confidence interval		p-Value
Colon	Morbidity	No vs. yes	2.17	1.69	2.78	<0.001
	Age	<50 vs. >79	4.45	1.98	9.99	<0.001
	pT-status	pT2 vs. pT4	2.40	1.11	5.20	0.026
	Risk factors	No vs. yes	1.73	1.13	2.62	0.011
	Adjuvant therapy	Yes vs. no	2.33	1.73	3.15	<0.001
	Gender	Female vs. male	1.46	1.14	1.87	0.002
Rectum	Morbidity	No vs. yes	1.80	1.38	2.34	<0.001
	Intraop. complication	Yes vs. no	1.81	1.21	2.69	0.004
	Age	<50 vs. >79	4.14	1.64	1.43	0.003
	pT-status	pT1 vs. pT4	2.26	1.22	4.17	0.009
	Risk factors	No vs. yes	1.89	1.27	2.83	0.002
	Gender	Female vs. male	1.34	1.03	1.75	0.029

HR, hazard ratio.

In rectal CA, a highly significant prognostic factor was determined: the occurrence of complications was associated with a 1,8-fold increased risk of death (HR: 1.80; 95-% CI: 1.38–2.34; p<0.001).

## Discussion

Considering the changes in MVR-associated incidence rates, the extended resection rates will possibly increase before and after the turn of the millennium [5, 13], [14], [15], [16]. The relative incidence of MVR listed in each study varied widely. The MVR rate was primarily dependent on the stages or depths of infiltration included. A scatter range of 5.5–100 % was reported in the literature [17, 18]. Taking into account the inclusion criteria, the MVR rate of 10 % reported in the present study corresponds to that reported in literature data [5, 6, 19].

However, is this MVR rate sufficient? For this purpose, the ratio of proven organ infiltration (pT4b) and MVR rates must be considered. Approximately 58.7 % of colon CA patients with pT4b tumors underwent MVR. Thus, the proportion of patients who did not undergo MVR despite the presence of infiltration was 41.3 %. In rectal CA, the MVR rate in patients with pT4b tumors was high (74.9 %), and 25.1 % of patients with existing malignant organ infiltration underwent a standard resection.

These relative frequencies of both tumor entities cannot be explained by palliative therapy intentions alone. Two decisive factors must be taken into account, as also documented in the literature. One is the intraoperative misinterpretation of the distinction between benign adhesion and infiltration [5]. On the contrary, Govindarajan et al. reported that the lack of surgical experience and the increased complication rates influenced the rate of MVR [20, 21]. Even though the relative frequencies mentioned are comparable with other studies, it is particularly true for colon carcinomas that more MVR should have been performed in everyday surgical practice [21, 22].

In both tumor entities, men were more frequently affected than women, but both the MVR groups had high proportions of women. This result is also reported by other studies, in some cases listing a sex ratio of 2:1 (women vs. men) [20]. Based on the SEER registry of the National Cancer Institute, the study conducted by Govindarajan et al. confirms the greater likelihood of MVR in women for both tumor entities [23].

This is not primarily associated with the biology of the respective tumors. Due to the different embryonic differentiation of the Müller and Wolff ducts and the later fetal descensus testis in the male sex, parenchymatous organs and hollow organs are located in the pelvis in women. A high number of organs increases the likelihood of MVR in women with CRC [23]. In addition, the organ structure influences the likelihood of infiltration. Thus, infiltration of the uterus is more likely than invasion of the dermal prostatic capsule [24]. In addition to these anatomical conditions, surgical aspects also influence MVR. Infiltrating rectal CA involving the prostate and urinary bladder is surgically challenging and associated with increased complication rates [16, 25, 26]. In the same tumor process in women, the uterus “shields the urinary bladder from the bowel,” which is why this organ is considered as a barrier. In comparison to the tumor-infiltrated urinary bladder, a tumor resection involving the female genitalia is less critical [20, 27].

These factors influence the outcome and can be represented with the multivariate Cox regression (MPA) data (Table 5). Men have a significantly higher risk of dying from one of the two carcinomas [28]. In a study conducted by Rosander et al., the risk of mortality in men with colon CA increased almost threefold (HR: 2.80; p<0.05) [4]. Govindarajan et al. chose the opposite calculation approach: according to this prospective study, women had a reduced probability of dying from colon or rectal CA [23].

In both nMVR and MVR groups, colon CA occurs within the seventh decade of life. However, rectal CA is common among young cohorts. After comparing our data with those in the literature, we found that extensively resected rectal CA patients are younger and that our study patients were older than those included in other studies. Nevertheless, studies with similar age distribution have been published [6, 29, 30]. The importance of age on outcome could be elaborated. Patients aged 79 years and older have a more than fourfold increased risk of death when MVR of colon CA is necessary (HR: 4.45; 95-% CI: 1.98–9.99; p<0.001). For rectal CA, the impact of this age group on survival is similar (HR: 4.14; 95-%CI: 1.64–10.43; p=0.003). The impact was estimated based on the observed survival rates. No age correction was made.

A comparison of studies confirms the influence of age on the rates of MVR [5, 15, 23, 25, 31]. Ike et al. were able to determine an increased risk of death among rectal CA patients aged 65 years and older in the multivariate Cox regression [32]. Darakhshan et al. calculated the significant effect of age (75 years and older) on survival in a colorectal study separately for both tumor forms (rectal CA – HR: 2.1; p<0.001/colon CA – HR: 3.2; p<0.001) [28].

An extension of the operation can only be demanded if this does not lead to a drastic increase in the complication rate or morbidity and lethality. There is disagreement among experts as to whether MVR leads to an increase in these rates. Although some studies reported no or moderate increase in the morbidity and mortality [3, 33], [34], [35], [36], other studies documented a significant increase [6, 37], [38], [39]. According to our own data, these rates were significantly higher among those who underwent MVR.

As already pointed out in other studies, a relatively high complication rate can be observed in patients with rectal CA [8, 40, 41]. Rectal surgery is a more demanding procedure than colon surgery, which may be due to the bony limitation of the pelvis. Additional radiochemotherapy procedures established today in the neoadjuvant setting are also influential in this regard [8, 9, 42, 43]. Another reason for the high complication rates is the (presumed) inclusion of total pelvic exenterations (TPE). High complication rates were reported in this “maximum variant” of MVR [20, 44], [45], [46]. In the present study, at least three organs were resected in 120 rectal CA patients. In this case, TPE must be assumed. A precise delimitation was not possible due to the specific study design.

As already mentioned, the patient population evaluated was older than that in other studies. This had a significant influence on the rate of complications. The study by Marusch et al. illustrated this aspect: if the morbidity of patients aged below 64 years remained 21.5 %, 41.2 % of it was in patients aged 80 years and older [10].

The significant effect of complications on survival is shown in Table 5. Morbidity was associated with a twofold higher probability of death in the multivariable analysis. In rectal CA, the significant influence of intraoperative complications on overall survival was also evident.

In contrast to the results of other studies, the study presented here showed an increase in the hospital lethality due to MVR in both tumor entities [14, 35, 47], [48], [49]. Kruschewski as well as Lehnert et al. detected an increased lethality in colon CA patients compared with the resected colon and rectal CA patients [5, 6]. The range of published rates in the literature review is 0–12 % [18, 34, 38, 47, 50, 51]. With a lethality of 4.9 and 3.8 % in the MVR group and 4.8 and 3.4 % in the matched-pair analysis group (colon CA and rectal CA, respectively), the data for both tumor entities are comparable with those of previously published studies [4, 9, 14, 27, 52].

The quality of a surgical intervention can be derived from the long-term data. In both carcinomas, it can be shown that compared to standard resections, the extended resections of the individual T-stages tend to be associated with reduced 5-YSR [25, 39, 51, 53, 54]. This statement also applies to the matched-pair analysis. Furthermore, no significant difference was observed in the 5-year-overall survival between the study group (MVR) and the control group (nMVR) (p=0.189) [14, 50]. For colon CA, these statements only apply proportionally. The 5-year-overall survival was significantly different between the MVR and nMVR groups. A significant difference was observed in the 5-year-overall survival in patients with pT4 tumors [5, 22, 33, 55, 56]. In addition, the determined 5-YSR was in accordance with that reported in the literature [3, 30, 33, 57].

Since a direct comparison of both tumor entities shows a favorable 5-year survival in rectal CA, a tendential survival advantage for patients with this tumor type can be postulated. Wasmann et al. already pointed this out in their study, suspecting a connection with the intraperitoneal location of the colon [58]. Other studies confirmed this difference in the 5-YSR [7, 28, 59].

Significant differences were found in the 5-year local recurrence rate (LRR) between the MVR group and the comparison group. In the matched-pair analysis, however, no significant difference was observed. In rectal CA, the 5-year-LRR of the study group (MVR) and the comparison group (nMVR) were even identical. The 5-year-LRR in patients who had undergone extended resections partially increased [22, 50, 60]. Due to the anatomical narrowness of the pelvis, a high 5-year – LRR in rectal CA would be conceivable and explainable, but this assumption is not confirmed by the present data, not even in the matched-pair analysis [60].

### Strengths

A major advantage of this study is the relatively large sample size. As this study focused on MVR, only unicentric data could be used for literature comparison. The multicenter data of 364 clinics presented here confirm the results of this comparison and correspond to literature data. Thus, it is also possible to make a rudimentary statement about the quality of care in the area. However, this broad-based approach also has disadvantages.

### Limitations

Feedback on recurrence is often inaccurate and has a limited coverage compared with the primary data, making it difficult to differentiate between local and distant events. Furthermore, the reasons for censoring can only be traced with much effort. Initial investigations included UICC stage IV as one of the reasons. However, evaluation of the most frequently resected organs of patients with stage-IV colon CA revealed robust evidence that organs (or their portions) were resected due to distant metastases and not due to infiltrations or adhesions. Furthermore, participating hospitals should use a uniform classification system (for example, the Clavien-Dindo classification) for better comparability of the complication rates.

### Outlook

The necessity of performing MVR also indicates the potential for tumor progression or a tendency toward progressive tumor growth in patients with a low depth of infiltration; hence, the abdominal surgeon should pay close attention to this issue with careful risk (benefit) assessment of the patient concerned and should take into account the invasiveness, potential complications, and lethality risk of surgical interventions. In these situations, surgery alone enables curation by R0 resection with a sufficient safety margin according to the established resection standards with adequate long-term survival as well as low local recurrence rates and is the decisive prognostic factor in this regard [61]. Continuous and further data analyses should be conducted to evaluate our results based on the indicated internal quality standards and to compare these results with those of other scientific studies using standardized systems of classifying surgical complications (for example, according to the Clavien–Dindo classification; refer to “Limitations” as well), nutrition, and quality of life aspects.

## Conclusions

MVR of colon or rectal CA is associated with increased morbidity and mortality risks (Table 6). Furthermore, the data collected show that patients undergoing extended resection (MVR) have an adequate long-term oncosurgical outcome (Table 6). However, compared with the conventionally resected group (nMVR), MVR tends to be associated with reduced 5-YSR (Table 6). This knowledge is important for the surgeon, as well as, in communicating with the patient. The present study underlines that MVR is not only justified but also must be demanded if indicated.

**Table 6:** Taking into account tumor type and type of surgical intervention, description of patient clientele, infiltration rate, morbidity and lethality as well as 5-year (yr)-overall survival, -tumor-free survival, and -local-recurrence rate.

		Colon						Rectum					
		MVR		nMVR		In total		MVR		nMVR		In total	
		n	%	n	%	n	p	n	%	n	%	n	p
Gender													

Men		720	46.4	7,605	54.1	8,325	<0.001	460	44.8	5,562	64.0	6,022	<0.001
Women		831	53.6	6,448	45.9	7,279		567	55.2	3,128	36.0	3,695	

Age													

Mean ± S_D_^a^		71.2 ± 11.5		71.5 ± 10.8			0.001	68.8 ± 11.0		68.4 ± 11.0			0.725

Infiltration													

pT4	Yes	602	82.2	423	30.1	602	<0.001	277	85.5	93	28.1	277	<0.001
	No	130	17.8	983	69.9	130		47	14.5	238	71.9	47	

Morbidity													

In total^a^			42.9		34.3		<0.001		46.3		37.2		<0.001

Lethality													

In total^a^		67	4.8	52	3.7	119	0.084	28	3.4	25	3.0	53	0.005

Overall survival													

5-yr-OAS^a^		54.1		62.4			0.004	60.5		63.6			0.189

Tumor-free survival													

5-yr-TFS^a^		47.5		57.4			<0.001	51.8		55.6			0.144

Local recurrence rate													

5-yr-LRR^a^		7.0		4.6			0.203	5.8		5.8			0.587

^a^Values of matched-pair analysis.
